# The Matthews correlation coefficient (MCC) should replace the ROC AUC as the standard metric for assessing binary classification

**DOI:** 10.1186/s13040-023-00322-4

**Published:** 2023-02-17

**Authors:** Davide Chicco, Giuseppe Jurman

**Affiliations:** 1 Institute of Health Policy Management and Evaluation, University of Toronto, 155 College Street, M5T 3M7 Toronto, Ontario Canada; 2 Data Science for Health Unit, Fondazione Bruno Kessler, Via Sommarive 18, 38123 Povo, Trento, Italy

**Keywords:** Matthews correlation coefficient, Receiver operating characteristic curve, ROC, Area under the curve, AUC, ROC AUC, Confusion matrix, Binary classification, Supervised machine learning, Data mining, Data science

## Abstract

Binary classification is a common task for which machine learning and computational statistics are used, and the area under the receiver operating characteristic curve (ROC AUC) has become the common standard metric to evaluate binary classifications in most scientific fields. The ROC curve has *true positive rate* (also called *sensitivity* or *recall*) on the *y* axis and false positive rate on the *x* axis, and the ROC AUC can range from 0 (worst result) to 1 (perfect result). The ROC AUC, however, has several flaws and drawbacks. This score is generated including predictions that obtained insufficient sensitivity and specificity, and moreover it does not say anything about *positive predictive value* (also known as *precision*) nor negative predictive value (NPV) obtained by the classifier, therefore potentially generating inflated overoptimistic results. Since it is common to include ROC AUC alone without precision and negative predictive value, a researcher might erroneously conclude that their classification was successful. Furthermore, a given point in the ROC space does not identify a single confusion matrix nor a group of matrices sharing the same MCC value. Indeed, a given *(sensitivity, specificity)* pair can cover a broad MCC range, which casts doubts on the reliability of ROC AUC as a performance measure. In contrast, the Matthews correlation coefficient (MCC) generates a high score in its $[-1; +1]$ interval only if the classifier scored a high value for all the four *basic rates* of the confusion matrix: sensitivity, specificity, precision, and negative predictive value. A high MCC (for example, MCC $=$ 0.9), moreover, always corresponds to a high ROC AUC, and not vice versa. In this short study, we explain why the Matthews correlation coefficient should replace the ROC AUC as standard statistic in all the scientific studies involving a binary classification, in all scientific fields.

**Supplementary Information:**

The online version contains supplementary material available at 10.1186/s13040-023-00322-4.

## The advantages of MCC over ROC AUC

**Binary classification.** A binary classification is a task where data of two groups need to be classified or predicted to be part of one of those two groups. Typically, the elements of one of the two groups are called negatives or zeros and the elements of the other group are called positives or ones. To evaluate the binary classification, researchers have introduced the concept of *confusion matrix*, a $2 \times 2$ contingency table where the positive elements correctly classified as positives are called true positives (TP), the negative elements wrongly classified as positive are called false positives (FP), the negative elements correctly classified as negatives are called true negatives (TN), and the positive elements wrongly classified as negatives are called false negatives (FN). When the predictions are binary, the evaluation involves a single confusion matrix. Many times, however, the predictions are real values in the [0; 1] interval. In such cases, a heuristic cut-off threshold $\tau = 0.5$ is used to map the real values into zeros or ones: predictions below $\tau$ are considered zeros, and the predictions equal or above $\tau$ are considered ones.

Caveat emptor: in this study, we refer to all the confusion matrix rates generated with cut-off threshold $\tau = 0.5$ for the confusion matrix, except ROC AUC which refers to all the possible cut-off thresholds, as we explain later. This choice of the threshold follows a well consolidated convention in the literature, and allows a fair comparison of the considerations presented hereafter with the outcome of most of the published references. When we write TPR $=$ 0.724, for example, we refer to a sensitivity value calculated when the confusion matrix cut-off threshold is $\tau = 0.5$. In the tables, we highlight this aspect by using the notation TPR$_{\tau = 0.5}$ rather than just TPR. However, in the body of this manuscript we decided to use the simple term TPR to make this study more readable.

Additionally, even if some scientific discoveries presented in this study are valid also for multi-class classification, we concentrated this study on binary classifications for space reasons. Analysis of multi-class classification rates [1–3] can be an interesting development for a future study.

**Confusion matrix rates.** The four categories of the confusion matrix, by themselves alone, do not say much about the quality of the classification. To summarize the outcome of the confusion matrix, researchers have introduced statistics that indicate ratios of the four confusion matrix tallies, such as *accuracy* and *F*_1_ *score*.

In a previous study [4], we defined *basic rates* for confusion matrices as the following four rates: sensitivity (Eq. 1), specificity (Eq. 2), precision (Eq. 3), and negative predictive value (Eq. 4).

1 $$\begin{aligned}&\text {true positive rate, recall, sensitivity, TPR} = \frac{\text {TP}}{\text {TP+FN}}\\&\text {(worst and minimum value 0; best and maximum value 1)}\nonumber \end{aligned}$$

2 $$\begin{aligned}&\text {true negative rate, specificity, TNR} = \frac{\text {TN}}{\text {TN+FP}}\\&\text {(worst and minimum value 0; best and maximum value 1)}\nonumber \end{aligned}$$

3 $$\begin{aligned}&\text {positive predictive value, precision, PPV} = \frac{\text {TP}}{\text {TP+FP}}\\&\text {(worst and minimum value 0; best and maximum value 1)}\nonumber \end{aligned}$$

4 $$\begin{aligned}&\text {negative predictive value, NPV} = \frac{\text {TN}}{\text {TN+FN}}\\&\text {(worst and minimum value 0; best and maximum value 1)}\nonumber \end{aligned}$$

A perfect classification, wherein all the positives are classified positives and all the negatives are classified negatives, means that all these four rates are equal to 1. Sensitivity and specificity can be seen as the ratio of correctly classified positives and negatives on the ground truth positives and ground truth negatives, respectively. Precision and negative predictive value, instead, can be interpreted as the ratio of correctly predicted positive elements made on all the positive predictions, and the ratio of all the rightly classified negative elements made on all the negative predictions.

Sensitivity and specificity are summarized in bookmaker informedness $\text {BM} = \text {TPR} + \text {TNR} -1$, while precision and negative predictive value are summarized in markedness $\text {MK} = \text {PPV} + \text {NPV} - 1$. Both BM and MK range in the [0; 1] interval with 0 meaning worst possible value and 1 meaning best possible score.

F$_1$ score (Eq. 5) and accuracy (Eq. 6), additionally, are common statistics which indicate respectively the ratio of true positives and true negatives over all the elements and the mean of precision and recall. F$_1$ score is actually a special case of the F$_\beta$ measure [5] with $\beta =1$.

5 $$\begin{aligned}&\mathrm {F}_{1}\text {score} = \frac{2 \cdot \text {TP}}{2 \cdot \text {TP} + \text {FP} + \text {FN}} = 2 \cdot \frac{\text {sensitivity} \cdot \text {precision}}{\text {sensitivity} + \text {precision}}\\&\text {(worst and minimum value 0; best and maximum value 1)}\nonumber \end{aligned}$$

6 $$\begin{aligned}&\text {accuracy} = \frac{\text {TP} + \text {TN}}{\text {TP} + \text {FN} +\text {TN} +\text {FP}}\\&\text {(worst and minimum value 0; best and maximum value 1)}\nonumber \end{aligned}$$

F$_1$ score and accuracy, although popular, can generate inflated overoptimistic results, especially on positively-imbalanced datasets [6].

As we explained in other studies [4, 6], the only rate that maximizes all the four basic rates is the Matthews correlation coefficient (MCC) (Eq. 7):

7 $$\begin{aligned}&\text {MCC} \; = \frac{\text {TP} \cdot \text {TN} - \text {FP} \cdot \text {FN}}{\sqrt{(\text {TP}+\text {FP})\cdot (\text {TP}+\text {FN})\cdot (\text {TN}+\text {FP})\cdot (\text {TN}+\text {FN})}}\\&\text {(worst and minimum value -1; best and maximum value +1)}\nonumber \end{aligned}$$

The MCC is a special case of the $\phi$ coefficient [7] for $2 \times 2$ confusion matrices: a +1 value corresponds to perfect classification; a value close to 0 corresponds to a prediction made by chance; and $-1$ corresponds to a perfectly opposite prediction, where all the negative samples were predicted as positive and vice versa [8]. Although it was first introduced in biochemistry [8], the MCC gained popularity in several scientific fields, including software defection prediction [9], recommender systems [10], pattern recognition [11], medicine [12], and affective computing [13], just to mention a few. Recently, the MCC has been proposed as one of the standard measures for biomedical image analysis validation by an international consortium of researchers working on that field [14].

In some previous studies, we argued that the MCC is more informative than confusion entropy [15], F$_1$ score [6], accuracy [6], balanced accuracy [4], bookmaker informedness [4], markedness [4], diagnostic odds ratio [16], Cohen’s Kappa [17], and Brier score [17].

The MCC is often scaled in the [0; 1] interval, so that it can have the same value range and meaning of the other statistical rates. We call this normalized coefficient *normMCC* (Eq. 8):

8 $$\begin{aligned}&\text {normMCC} \; = \frac{\text {MCC} + 1}{2}\\&\text {(worst and minimum value = 0; best and maximum value = 1)}\nonumber \end{aligned}$$

The key asset of the MCC is that its high value always corresponds to high values for each of the four confusion matrix *basic rates*: sensitivity, specificity, precision, and negative predictive value [4]. No other widespread confusion matrix statistic has this feature, although recently novel measures exploiting such property has been proposed [18, 19].

**The ROC curve.** Even if using a heuristic $\tau$ threshold for confusion matrices is a common practice in machine learning and computational statistics, it has the flaw of employing an arbitrary value. One might ask: “Why 0.5? Why not 0.4 or 0.6?”, and it would be a legitimate question. Some researchers employ an approach called *reclassification* where multiple cut-off thresholds are tested [20], but the arbitrariness of these choices still remains.

To avoid picking a specific arbitrary threshold, researchers introduced evaluation curves, that are depicted by computing statistics on all the possible confusion matrices of a binary classification. To generate these curves, each gold standard element of the test set is sorted increasingly and then used a cut-off threshold for a confusion matrix: predicted values above or equal to that threshold are mapped into 1s, while predicted values below that threshold are mapped into 0s. This way, the evaluation method computes a specific confusion matrix for each element of the test set gold standard; if the test set contains N elements, then N confusion matrices are computed. The rates computed on these N confusion matrices are then employed as axes to generate points in curves such as the ROC curve [21].

The most common evaluation curve worldwide is the receiver operating characteristic curve (ROC) [22], an evaluation technique originally introduced for operators of military radar receivers during the Second World War. In the 1940s, radar operators in the United States army had to decide whether a blip on the screen indicated an enemy target, an allied ship, or just noise [23], and that is how and when the concept of ROC curve was introduced. The *receiver* was the soldier or army employee who was *operating* in real time to analyze radar images. He had to collect the information from the radar images, called *characteristics*, which is how the name *receiver operating characteristics* started.

In the early 1970s, Lee Lusted proposed the adoption of the ROC curves as diagnostic performance tool in radiology [24]. Since then, researchers began using the ROC curve as a binary classification assessment tool in several fields, especially in medicine, biostatistics, epidemiology, healthcare [25–27], and bioinformatics [28], until it became perhaps the most used metric to assess binary classification tests in any scientific field [29–31].

Nowadays, it is hard to find a binary classification study in biomedical informatics which does not include results measured with ROC curves. To give an idea, to date the scientific articles present in Google Scholar [32] which contain the “ROC curve” keyword total approximately 612 thousand. The same search made for “F1 score” led to 101 thousand articles, while for “Matthews correlation coefficient” it found 20 thousand manuscripts to date: the number of studies that mention the ROC AUC is approximately 30 times the number of articles which refer to Matthews correlation coefficient.

The ROC curve has *true positive rate* (also called *sensitivity* or *recall*) on the *y* axis and false positive rate on the *x* axis. The area under the ROC curve (ROC AUC, also known as *c statistic*) is one of the most common statistics used in scientific research to assess binary classifications, and can range from 0 (worst result) to 1 (perfect result) [30]. The ROC AUC, however, has multiple flaws and disadvantages [33–37], which have emerged especially in medical studies [33, 34, 38–43]: in particular, the ROC AUC is computed by taking into account the portions of ROC space where the classifications generated at least one sufficient rate between sensitivity and specificity and the portions of ROC space where both sensitivity and specificity are insufficient (Fig. 3a). We consider a score *insufficient* if its value is below 50% of correctness in its interval (in this case, TPR < 0.5 and TNR < 0.5).

Moreover, the ROC AUC does not say anything about precision and negative predictive value. The ROC curve, in fact, has sensitivity on the *y* axis and false positive rate on the *x* axis. Since false positive rate corresponds to ${1 - \text {specificity}}$, the area under the ROC curve is symmetrical on the *y* axis with the sensitivity-specificity curve. In particular, a high ROC AUC always corresponds to at least one high rate between sensitivity and specificity: as we can notice in the ROC example in Fig. 3a, a ROC curve always starts at the point with coordinates $(TNR,TPR) = (1,0)$ in the bottom left corner, and finishes at the point $(TNR,TPR) = (0,1)$ in the top right corner. Since ROC AUC goes from 0 to 1, the Cartesian distance of each point of the ROC from the plot origin can range only from 0 to $\sqrt{2} = 1.414$. In the case of maximum perfect AUC $=$ 1.000, the ROC curve includes the point $(TNR,TPR) = (1,1)$ on the top left corner, which corresponds to perfect maximum sensitivity and perfect maximum specificity. In a ROC curve, sensitivity (Eq. 1) and specificity (Eq. 2) are proportionally anti-correlated: if sensitivity increases, specificity decreases, or vice versa.

In our Fig. 3a example, we have sensitivity $=$ 0.724 and specificity $=$ 0.789 when the cut-off threshold is $\tau = 0.5$.

In non-perfect ROC curves, such as the Fig. 3a example, we can see that the points in the bottom left quadrant correspond to low sensitivity and high specificity, the points in the top right quadrant correspond to low specificity and high sensitivity, and the points in the top left quadrant correspond to both high specificity and high sensitivity.

**Fig. 1:**
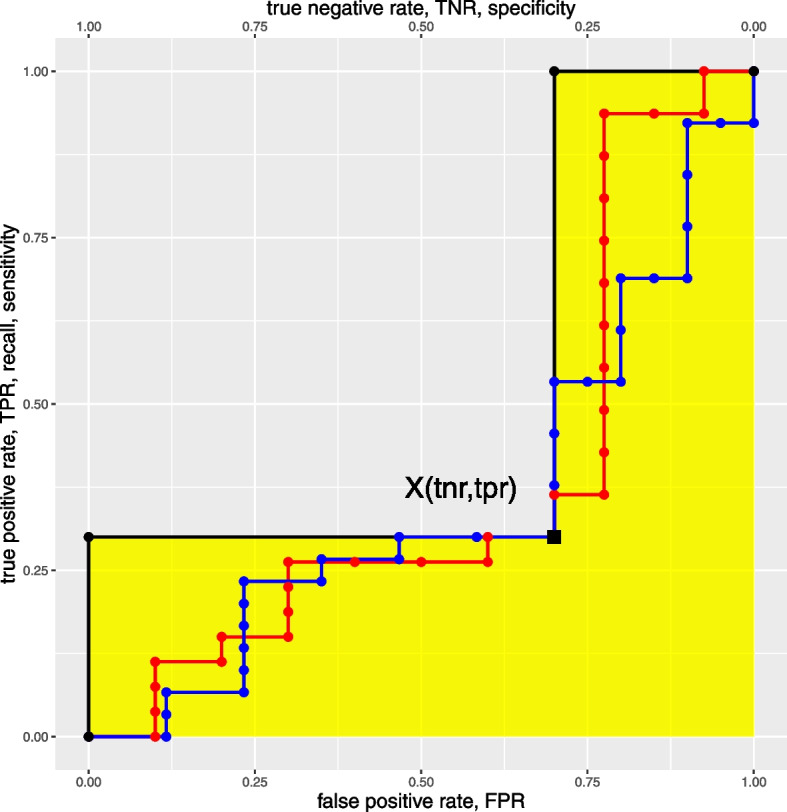
ROC curves passing through the point X. Among all ROC curves passing through the point $X(\text {tnr},\text {tpr})$ (with *x* coordinate expressed in terms of the secondary TPR axis), the black one is the curve maximising the AUC area, marked in yellow. The red dotted curve and the blue dotted curve represent two random ROC curves that pass through the highlighted *X* black point

**Relationship between ROC AUC and (TNR,TPR) points. **Consider the point *X* in the ROC space with coordinates $(\text {fpr},\text {tpr})$. For clearness’ sake, we use the alternative formulation $X(\text {tnr},\text {tpr})$, using the reverse x axis true negative rate, complement to 1 to the original FPR axis, so that $\text {tnr}=1-\text {fpr}$. This is graphically represented in Fig. 1.

Then, by construction, among all ROC curves having *X* as a point, the ROC maximising the ROC AUC can be built on the 5 points $\{(1,0), (0, \text {tpr}),X,(\text {tnr},1), (0,1)\}$ and the corresponding ROC AUC has value $(1-\text {tnr})\cdot \text {tpr}+\text {tnr} = \text {tpr}+\text {tnr} - \text {tpr}\cdot \text {tnr}$.

This yields that, for a ROC curve having area under the curve $\alpha$, all the points of the curve must satisfy the condition in Eq. 9:

9 $$\begin{aligned} \text {TNR}+\text {TPR}-\text {TNR}\cdot \text {TPR} < \alpha \ . \end{aligned}$$

Given that TNR, TPR, and $\alpha$ must be in the [0; 1] range, Eq. 9 is satisfied by all couples of coordinated lying above the upper arm of the equilateral hyperbole $\text {TNR}+\text {TPR}-\text {TNR}\cdot \text {TPR}-\alpha =0$ in a (regularly oriented) Cartesian plane with axes TNR and TPR.

**The assets of a high ROC AUC.** We define a ROC AUC “high” if it is greater than ${\pi }/{4} \simeq 0.785$. Geometrically, this value corresponds to the AUC of the ROC curve coinciding with the quarter of circle of radius 1, centered in $(\text {TNR},\text {TPR})=(0,0)$. By definition, all the points of this ROC curve satify the half semicircle equation $\text {TNR}^2+\text {TPR}^2=1$. When all points of a ROC curve lie outside such circle, clearly the corresponding ROC AUC is larger than ${\pi }/{4} \simeq 0.785$: in terms of coordinates, this is equivalent to say that at least one of the two coordinates $(\text {TNR},\text {TPR})$ is greater than ${\sqrt{1/2}} \simeq 0.71$. Note that the point *p*, intersection of the circle with the top-left and bottom-right diagonal of the plane, has exactly coordinates $\left( {\sqrt{1/2}},{\sqrt{1/2}}\right)$ . Thus, this is a sufficient condition for a ROC curve to yield a high AUC: all the aforementioned considerations are visually represented in Fig. 3b.

A necessary condition can be drawn by solving Eq. 9 for $\alpha ={\pi }/{4} \simeq 0.785$:

$$\begin{aligned} \text {TNR}+\text {TPR}-\text {TNR}\cdot \text {TPR}\ge \frac{\pi }{4}\ ,\text { with } 0\le \text {TNR},\text {TPR}\le 1\ . \end{aligned}$$

Solving in one of the variables, we obtain that the equation is satisfied by:

$$\begin{aligned} \left\{ \begin{array}{ll} \frac{\text {TPR} \ge \pi -4\cdot \text {TNR}}{4-4\cdot \text {TNR}} &{} \text {for}\ 0 \le \text {TNR} < \frac{\pi }{4} \\ \forall \ \text {TPR}\in [0,1] &{} \text {otherwise}\ . \end{array}\right. \end{aligned}$$

**Fig. 2:**
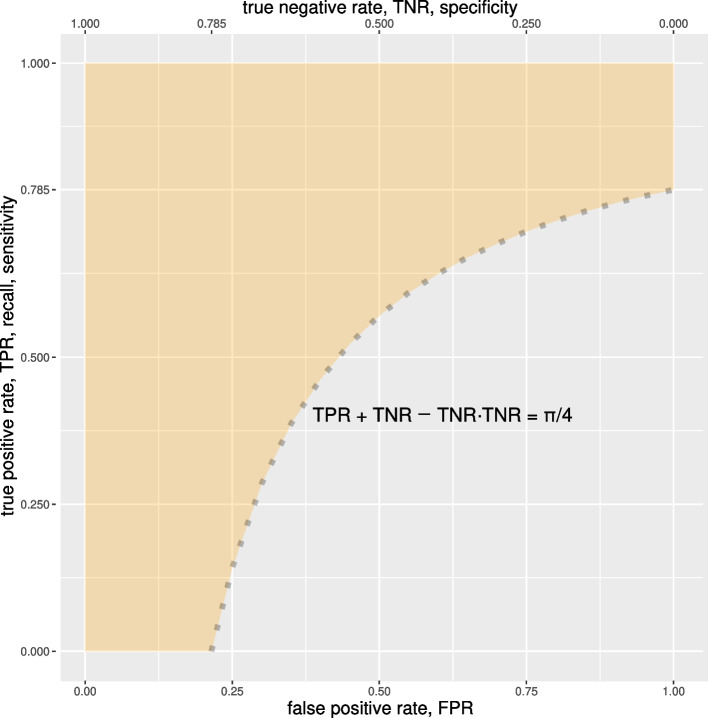
Solution of Eq. 9 for AUC $={\pi }/{4} \simeq 0.785$. The points within the yellow area are all the possible (*TNR*, *TPR*) points when the ROC AUC has value 0.785. Please notice that the ROC curve with $AUC = 0.785$ is not represented here. The black-dotted curve depicted here is one of the boundaries of the yellow area

Since the equation is symmetric in the two variables, the same relation holds when swapping TPR and TNR. A visual representation of the solution space is shown by the yellow shaded area in Fig. 2, while a summarizing table with numerical values can be found in Table 1.

Finally, for ROC AUC values larger than ${\pi }/{4}$, the solution space is similar, but clearly narrower, since the curved line of Fig. 2 would be translated towards the top-left angle of the Cartesian plane.

**Table 1:** Numerical approximation of some landmark values of $(\text {TNR},\text {TPR})$ yielded by a high ROC AUC of 0.785, that approximates $\pi /4$. For example, if TNR is 0.35, then TPR must be greater or equal to 0.670. Due to the symmetric nature of the necessary condition Eq. 9, the relation between the two rates $\text {TNR}$ and $\text {TPR}$ holds when swapped. $TNR \ge 0.00$ means that TNR can have any value in the [0; 1] range and $TPR \ge 0.00$ means that TPR can have any value [0; 1] range. Please notice that the half semicircle ROC represented by the blue line in Fig. 3b has AUC $= \pi /4 \simeq 0.785$, but there are several other ROC curves with the same AUC

situation when ROC AUC = 0.785					
if TNR $=$	then TPR $\ge$	if TNR $=$	then TPR $\ge$	if TNR $=$	then TPR $\ge$
0.00	0.785	0.35	0.670	0.70	0.285
0.05	0.774	0.40	0.642	0.75	0.142
0.10	0.762	0.45	0.610	0.80	0.000
0.15	0.748	0.50	0.571	0.85	0.000
0.20	0.732	0.55	0.523	0.90	0.000
0.25	0.714	0.60	0.463	0.95	0.000
0.30	0.693	0.65	0.387	1.00	0.000

**Fig. 3:**
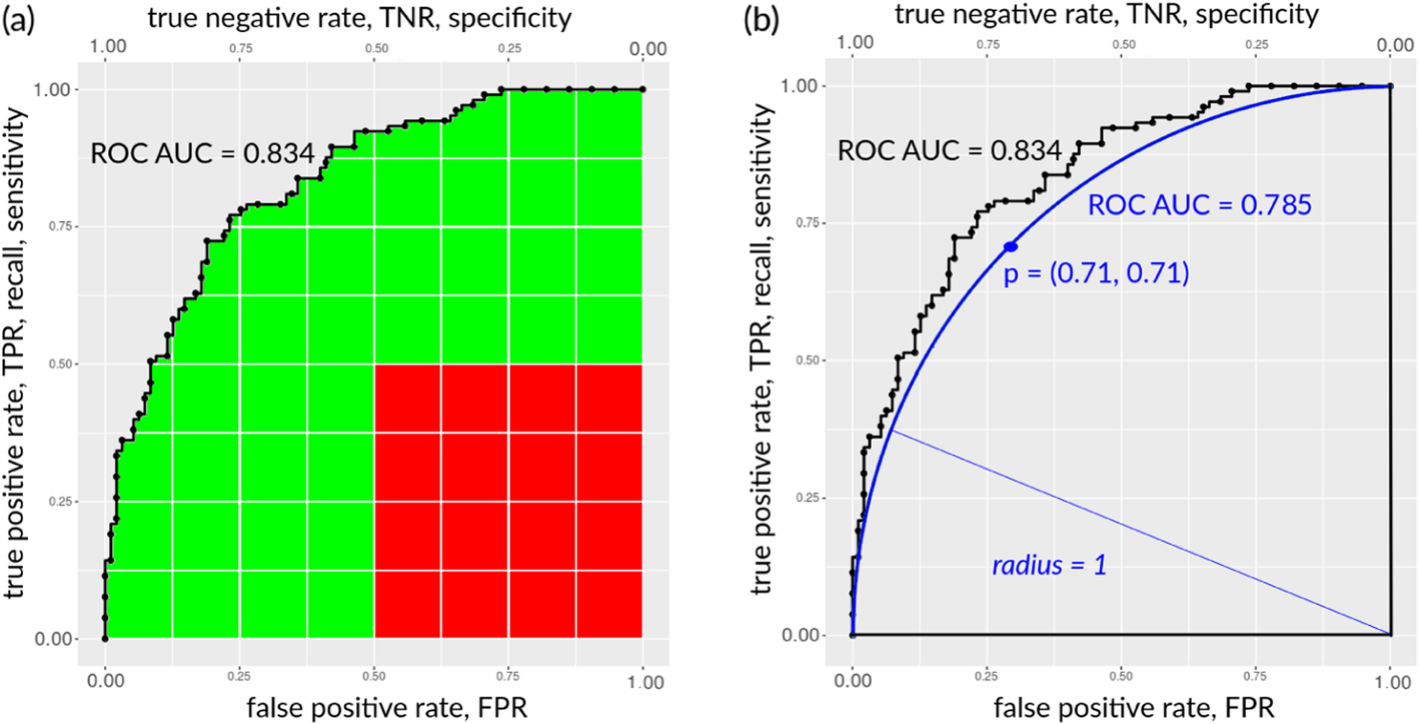
Example of ROC curve with area under the curve. **a** plot: This illustrative example contains a ROC plot having AUC $=$ 0.834 that indicates a good performance in the [0; 1] interval where 0 indicates completely wrong classification and 1 indicates perfect prediction. True positive rate, sensitivity, recall, $TPR = TP / (TP + FN)$. False positive rate, $FPR = FP / (FP + TN) = 1 - specificity$. The AUC consists of both the green part and the red part of this plot. As highlighted by Lobo and colleagues [33], the calculation of the AUC is done by considering portions of the ROC space where the binary classifications are very poor: in the ROC space highlighted by the red square, the sensitivity and sensitivity results are insufficient (TPR < 0.5 and FPR $\ge$ 0.5). However, this red square of bad predictions, whose area is $0.5^2 = 0.25$, contributes to the final AUC like any other green portion of the area, where sensitivity and/or sensitivity result being sufficient instead. This red square represents 30% of the AUC $=$ 0.834 and 25% of the whole maximum possible AUC $=$ 1.000. How is it possible that this red portion of poor classifications contribute to the final AUC like any other green part, where at least one of the two axis rates generated good results? We believe this inclusion is one of the pitfalls of ROC AUC as a metric, as indicated by Lobo and colleagues [33] and one of the reasons why the usage of ROC AUC should be questioned. **b** plot: The same ROC curve with the half semicircle having $AUC = \pi /4 \simeq 0.785$. Each point of the blue curve has $radius = 1$ and centre in $(TPR,TNR) = (0,0)$. Point *p*: point with $(TPR,TNR) = (\sqrt{\frac{1}{2}},\sqrt{\frac{1}{2}}) \simeq (0.71,0.71)$

More precisely, if the Cartesian distance between each point of the ROC curve and the bottom right corner point $(TNR,TPR) = (0,0)$ is always equal to 1, we have a half semicircle with area $\pi / 4 \simeq 0.785$ (Fig. 3b). If each distance is always 1, then $\sqrt{TPR^2 + TNR^2}$ must be equal to 1, too. If both TPR and TNR can range only in the [0; 1] intervals, then $TPR = \sqrt{1 - TNR^2}$ and $TNR = \sqrt{1 - TPR^2}$.

TNR and TPR have the same value only for the $(TNR,TPR) = (\sqrt{\frac{1}{2}},\sqrt{\frac{1}{2}}) \simeq (0.71,0.71)$ point *p* in Fig. 3b, because $TPR= \sqrt{1 - TNR^2} = \sqrt{1 - 0.71^2} = \sqrt{1 - 0.5} = \sqrt{0.5} = 0.71$ and $TNR = \sqrt{1 - TPR^2} = \sqrt{1 - 0.71^2} = \sqrt{1 - 0.5} = \sqrt{0.5} = 0.71$.

This means that, if an ROC AUC is greater than or equal to 0.785 and all the points are above or on the half semicircle with radius $=$ 1 and centre in the bottom-left corner point (TNR,TPR) $=$ (0,0), all the points of its ROC curve have both sensitivity in the [0.71; 1] interval or specificity in the [0.71; 1] interval. In other words, having a high ROC AUC means having at least TPR $=$ 0.71 or at least TNR $=$ 0.71, or higher values for both of them.

We represented the half semicircle ROC with radius $=$ 1 and centre in the bottom-left corner $(TNR,TPR) = (0,0)$ with the blue line in Fig. 3b.

To summarize, in any case with a high ROC AUC and all the curve points on or above the half semicircle ROC, at least one rate between specificity and sensitivity is high.

We can therefore update the recap table of the meanings of the confusion matrix summarizing rates (originally presented in Table 4 of [4]) in Table 2.

**Table 2:** Recap of the correlations between the confusion matrix summarizing metrics and the basic rates. #: integer number. MCC: Matthews correlation coefficient (Eq. 7). BA = balanced accuracy $= (TPR + TNR) / 2$. BM = bookmaker informedness $= TPR + TNR - 1$. MK = markedness $= PPV + NPV - 1$. F$_1$ score: harmonic mean of TPR and PPV (Eq. 5). Accuracy: ratio between correctly predicted data instances and all data instances (Eq. 6). We call “basic rates” these four statistics: TPR, TNR, PPV, and NPV. We calculate MCC, BA, MB, MK, F$_1$ score, accuracy, TPR, TNR, PPV, and NPV here with cut-off threshold $\tau = 0.5$: real-valued predictions greater or equal to 0.5 are mapped into 1s, and real-valued predictions smaller than 0.5 are mapped into 0s. The ROC AUC, instead, refers to all the possible cut-off thresholds, as per its definition. We published an initial version of this table as Table 4 in the [4] article under the Creative Commons Attribution 4.0 International License

scenario	condition of basic rates (with ${\tau = 0.5}$)	# guaranteed high basic rates
high MCC$_{\tau = 0.5}$	high TPR, TNR, PPV, and NPV	4
high BA$_{\tau = 0.5}$	high TPR, TNR, and at least one of PPV and NPV	3
high BM$_{\tau = 0.5}$	high TPR, TNR, and at least one of PPV and NPV	3
high MK$_{\tau = 0.5}$	high PPV, NPV, and at least one of TPR and TNR	3
high F$_1$ score$_{\tau = 0.5}$	high PPV and TPR	2
high accuracy$_{\tau = 0.5}$	high TPR and PPV, or high TNR and NPV	2
high ROC AUC$_{\tau = all}$ with all points above half semicircle ROC	high TPR and TNR, or at least one of TPR and TNR	1

**What a high ROC AUC does not say.** However, the ROC curve and its AUC provide no information about precision and negative predictive value. A classifier might generate a high ROC AUC of 0.9, with low precision of 0.12 and low NPV of 0.3. If a researcher decided to look solely at the ROC AUC and forget about all the other rates, as often happens, they might wrongly believe that their classification was very good, when in reality it was not. Conversely, a high value of the Matthews correlation coefficient, always indicates a high value for each of the four basic rates, eliminating the risk of overoptimistic or inflated outcomes.

A few other studies compared the MCC and the ROC in the past [11, 44], but they were not focused on the four basic rates that we use here. In the past, some researchers presented variants of ROC curves (cost curve [45], summary ROC curve [46], concentrated ROC curve [47], total operating characteristic curve [48], partial ROC curve [49–52], partial ROC AUC index [53], restricted ROC curve [54], uniform AUC [55], and not proper ROC curve [56]), but all of them share the same drawback with the original ROC curve: they do not provide any information about precision and negative predictive value obtained during the classification.

## The MCC-ROC AUC relationship

The analytical comparison between MCC and ROC AUC values for a classifier is hardly justifiable mathematically due to the intrinsic different nature of the two performance measures. Furthermore, it is straightforward to see that the same ROC AUC can be associated to deeply diverse ROC curves (and, as such, classifiers), thus covering a broad range of possible MCC values. Even a single given point in the ROC space can yield a wide span of MCC values, as shown in what follows, where we investigate the mathematical intertwining between MCC and ROC AUC, to show the wide mutual variability preventing the existence of a direct relation linking the two measures suitable for an analytical analysis.

### MCC and ROC

As a first step, we study the connection between points in the ROC space and the corresponding MCC. Despite the fact that MCC is generally acknowledged as robust against imbalanced datasets, this does not yield that MCC is independent of the class ratio. Actually, as shown by the MACQ Consortium [57], if we introduce the prevalence $p=\frac{\text {TP}+\text {FN}}{\text {TP}+\text {TN}+\text {FP}+\text {FN}}$ as the ratio of the actual positive samples over the total number of samples, by definition specificity (TNR) and sensitivity (TPR) do not depend on *p*, while MCC does.

Such dependence is even non-linear, as evidenced by the following formula:

10 $$\begin{aligned} \text {MCC}_{\text {TNR},\text {TPR}}(p) = \frac{\text {TNR}+\text {TPR}-1}{\sqrt{\left( 1-\text {TNR}+\frac{p}{1-p}\ \text {TPR} \right) \left( 1-\text {TPR}+\frac{1-p}{p}\ \text {TNR} \right) }}\ . \end{aligned}$$

the aforementioned equation is thus positive for $\text {TNR}+\text {TPR} > 1$ and negative otherwise, and it is antisymmetric for taking rates’ complement to 1: $\text {MCC}_{\text {TNR},\text {TPR}}(p)=-\text {MCC}_{1-\text {TNR},1-\text {TPR}}(p)$.

**Fig. 4:**
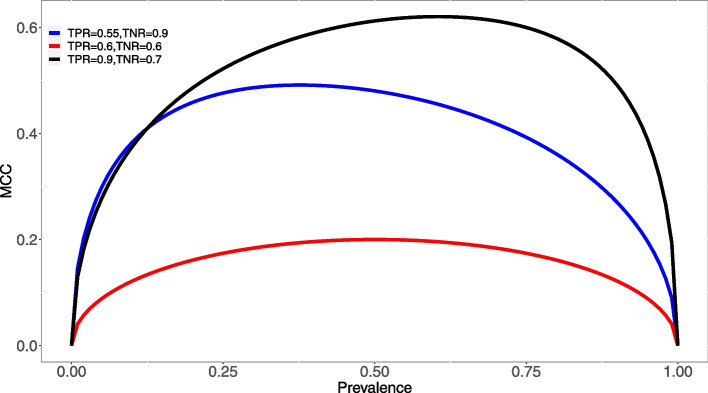
Plots of $\text {MCC}_{\text {TNR},\text {TPR}}(p)$ for different values of $\text {TNR}$ and $\text {TPR}$ with $\text {TNR}+\text {TPR} > 1$. The behaviour of MCC as a function of the prevalence *p* depends on the particular pair $(\text {TNR},\text {TPR})$; the curve tends to be more symmetric when values of $\text {TNR}$ and $\text {TPR}$ are similar, and MCC values are high when $\text {TNR}$ and $\text {TPR}$ are high. In the current plot we show three examples: one symmetric with low $\text {TNR}$ and $\text {TPR}$ values (red line), and two asymmetric curves, the former where both rates are high (black) and the latter where one one rate is high (blue). Due to the symmetry in the $\text {MCC}_{\text {TNR},\text {TPR}}(p)$ equation, we can restrict the display to the case $\text {TNR}+\text {TPR} > 1$. Finally, the non-linearity of the same equation prevents from predicting more precise features of the MCC behaviour in terms of $p,\text {TNR},\text {TPR}$

Moreover, MCC is symmetric for swapping classes and sensitivity and specificity $\text {MCC}_{\text {TNR},\text {TPR}}(p)= \text {MCC}_{\text {TPR},\text {TNR}}(1-p)$; furthermore, for extremely unbalanced dataset we have

11 $$\begin{aligned} \lim _{p\rightarrow 0} \text {MCC}_{\text {TNR},\text {TPR}}(p) = \lim _{p\rightarrow 1} \text {MCC}_{\text {TNR},\text {TPR}}(p) = 0\ , \end{aligned}$$

for any value of sensitivity and specificity. In Fig. 4 we plotted several $\text {MCC}_{\text {TNR},\text {TPR}}(p)$ curves as functions of the prevalence *p* for different values of specificity (TNR) and sensitivity (TPR). In view of the aforementioned antisymmetry, we considered only the case $\text {TNR}+\text {TPR} > 1$.

For a given pair $(\text {TNR},\text {TPR})$, the extreme value ${\bar{M}=\max _p|\text {MCC}_{\text {TNR},\text {TPR}}(p)|}$ is attained for $p=\bar{p}$, unique solution of the equation $\frac{\text {d}}{\text {d}p} \text {MCC}_{\text {TNR},\text {TPR}}(p)=0$. Defining $\lambda =\sqrt{\frac{\text {TPR}(1-\text {TPR})}{\text {TNR}(1-\text {TNR})}}$, then $\bar{p}=\frac{1}{\lambda +1}$ and $\bar{M}= \frac{\text {TNR}+\text {TPR}-1}{\sqrt{\left( 1-\text {TNR}+\lambda ^{-1}\ \text {TPR} \right) \left( 1-\text {TPR}+\lambda \ \text {TNR} \right) }}$.

**Fig. 5:**
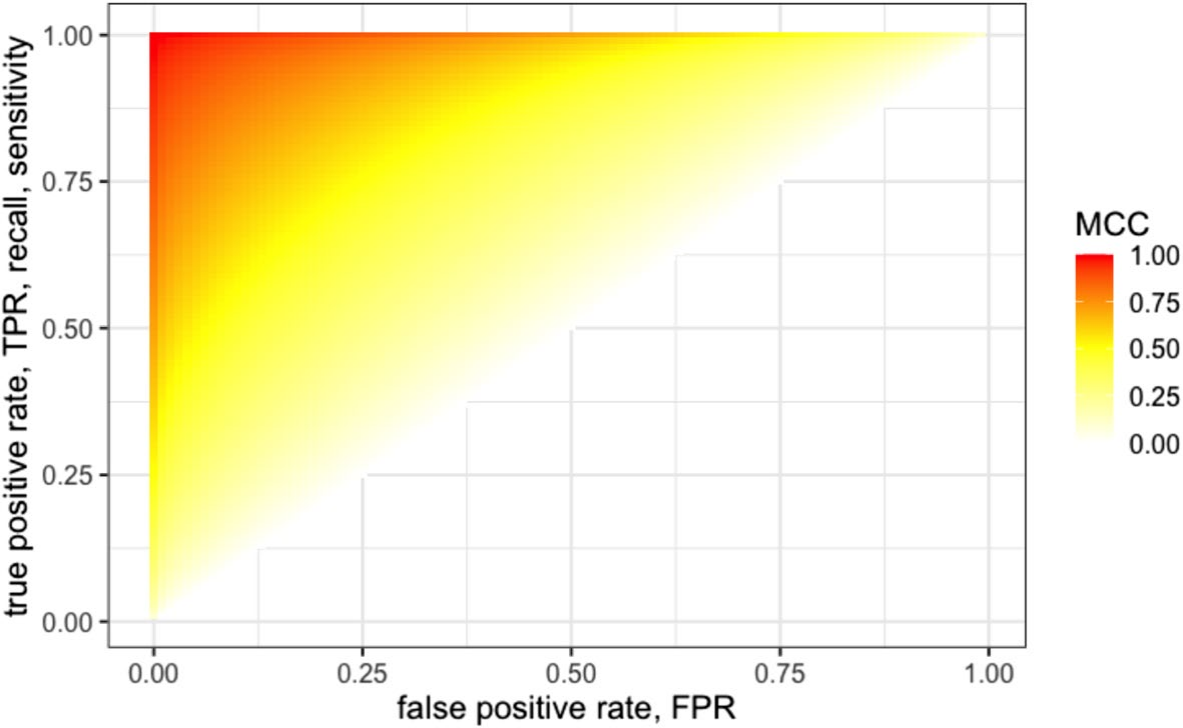
Heatmap of $\bar{M}$ for the $\text {TNR}+\text {TPR} > 1$ half of the ROC space. To get a global overview of the $\bar{M}$ values as a function of $\text {TNR}$ and $\text {TPR}$ we display a heatmap representation using a yellow to red palette which highlights the non-linear behaviour of the mapping, as evidenced by the curved isolines. As a straightforward consideration, $\bar{M}$ achieves high values only when both $\text {TNR}$ and $\text {TPR}$ are high: if one of the two rates is low, $\bar{M}$ values are bounded into a medium range. As in the previous plot, due to the symmetry in the $\text {MCC}_{\text {TNR},\text {TPR}}(p)$ equation, we can restrict the display to the case $\text {TNR}+\text {TPR} > 1$

Thus, for a point in the ROC space, defined by TPR and TNR, the corresponding value of MCC can vary (in the case $\text {TNR}+\text {TPR} > 1$) between 0 and $\bar{M}$: in Fig. 5 we showed the heatmap of $\bar{M}$ for the upper triangular half of the ROC space.

To provide a graphical representation of the situation we can use the Confusion Tetrahedron [16], a novel visualization tool able to behaviour of a binary classification metric on the full universe of the possible confusion matrices, by using the concept of equivalence class. Consider the pair $(x,y)=(\text {FPR},\text {TPR})= \left( \frac{\text {FP}}{\text {FP+TN}}, \frac{\text {TP}}{\text {TP+FN}}\right)$: since sensitivity, specificity and MCC are invariant for the total number of samples of binary dataset, each CM entry can be divided for the sum of the entries, so that all the four values TP, TN, FP and FN are limited in the unit range [0; 1]. As an example, all the three confusion matrices $$\left( \begin{array}{cc} 50 &{} 20\\ 40 &{} 30 \end{array}\right)$$ , $$\left( \begin{array}{cc} 35 &{} 14\\ 28 &{} 21 \end{array}\right)$$ and $$\left( \begin{array}{cc} 65 &{} 26 \\ 52 &{} 39 \end{array}\right)$$ share the same representative matrix $$\left( \begin{array}{cc} 0.3571429 &{} 0.1428571 \\ 0.2857143 &{} 0.2142857 \end{array}\right)$$.

**Fig. 6:**
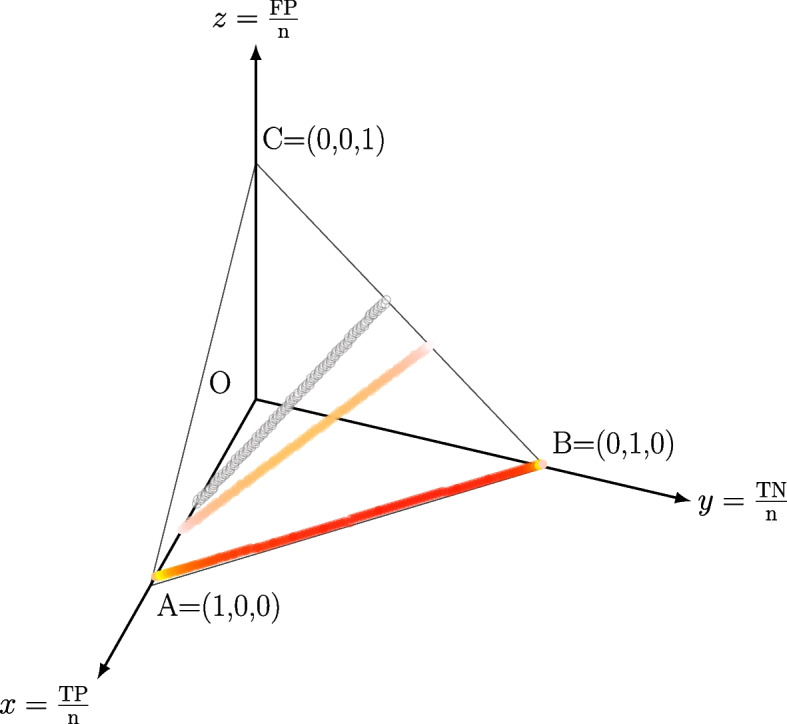
Three sets of confusion matrices sharing the same sensitivity and specificity in the Confusion Tetrahedron. Bottom line, $(x,y)=(0.01,0.95)$, top line $(x,y)=(0.55,0.56)$, middle line $(x,y)=(0.4,0.7)$. Colors of points determined by MCC value, according to the gradient in Fig. 5. *n* is the sum of all entries of the confusion matrices

Given one of such representative matrix $$\left( \begin{array}{cc} \text {TP} &{} \text {FN}\\ \text {FP} &{}\text {TN}\end{array}\right)$$, all the multiple matrices $$\left( \begin{array}{cc} n\cdot \text {TP} &{} n\cdot \text {FN}\\ n\cdot \text {FP} &{}n\cdot \text {TN}\end{array}\right)$$ will share the same sensitivity, specificity and MCC for any $n\in \mathbb {N}$. As a first observation, the pair $(x,y)=(\text {FPR},\text {TPR})$ does not univocally identify a CM. For instance, the two confusion matrices $$\left( \begin{array}{cc} 0.4 &{} 0.2\bar{6}\\ 0.0\bar{3} &{} 0.3\end{array}\right)$$ and $$\left( \begin{array}{cc} 0.2 &{} 0.1\bar{3}\\ 0.0\bar{6} &{} 0.6\end{array}\right)$$ share the same pair $(x,y)=(\text {FPR},\text {TPR})=(0.1,0.6)$. In detail, the four entries have ranges $0<\text {TP}<y$, $0<\text {FN}<1-y$, $0<\text {TN}<1-x$, $0<\text {FP}<x$, and all the confusion matrices sharing the same pair (*x*, *y*) are generated within these bounds by the linear relation $\text {TP}/y+\text {FP}/x=1$. We provide a visual example of a set of confusion matrices corresponding to the same (*x*, *y*) point in the Confusion Tetrahedron space in Fig. 6.

**Fig. 7:**
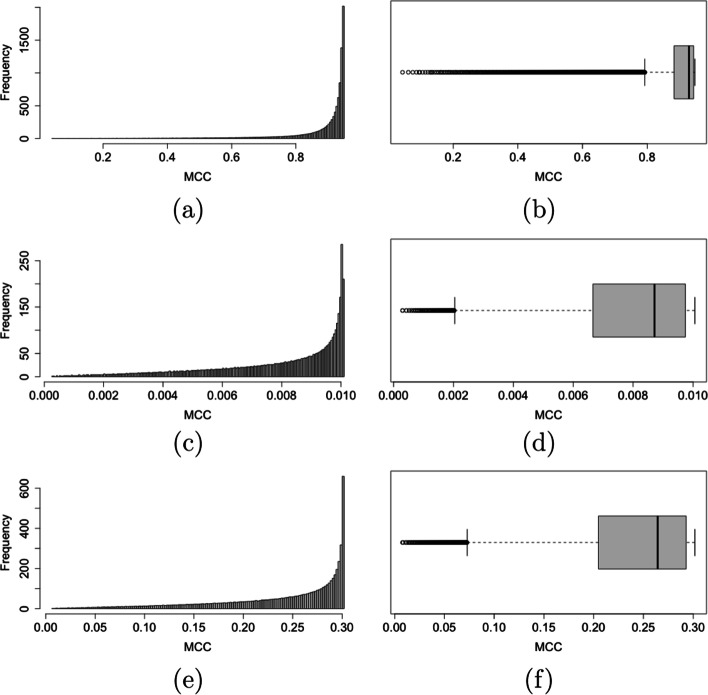
Histogram (left) and box-and-whiskers (right) plots of MCC values for the three (*x*, *y*) points in the ROC space (0.01,0.95) (**a**,**b**), (0.55,0.56) (**c**,**d**), and (0.4,0.7) (**e**,**f**)

Finally, distribution of MCC values for a given (*x*, *y*) point in the ROC space is shown in Fig. 7: as the two most relevant features, the distribution is always heavily left skewed, and its shape mainly depends on the value of $\vert y-x\vert$.

All the results in this section highlight the broad variability affecting the relationship between a point in the ROC space and the associated MCC value, encompassing disparate situations in classification tasks and leaving room for deeply diverse interpretation of the same binary classification model.

### ROC AUC and MCC dynamics

**Fig. 8:**
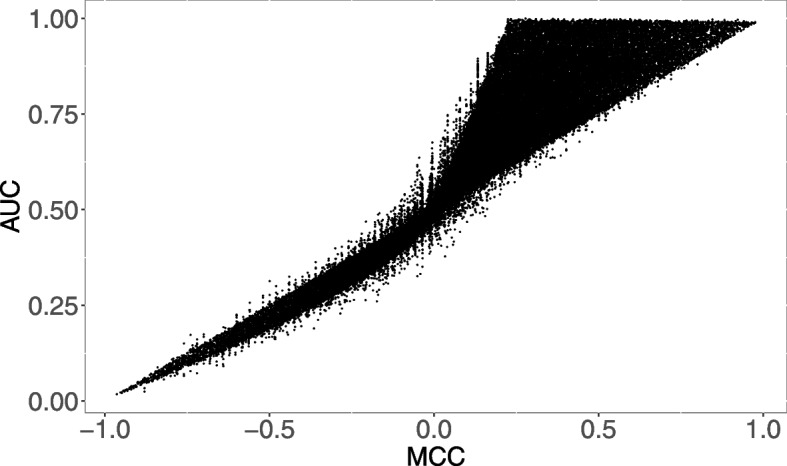
Scatterplot of ROC AUC versus MCC values for 70 thousand simulated binary classification tasks

The results of the previous section hit even harder the behavior of the dynamics of ROC AUC versus MCC values: here we investigate the ROC AUC versus MCC relationship at a global level: being the analytical approach unfeasible, we show a landscape of simulations framing the issue. In particular, we simulated about 70 thousand binary classification tasks with number of samples randomly selected in the set $\{10^k:k\in \mathbb {N},2\le k\le 6\}$ and prevalence randomly extracted in the interval [0.05, 0.5]. We used the values of ROC AUC and MCC for these simulations as the axes for the scatterplot reported in Fig. 8.

Although there is quite a reasonably aligned trend between the two measures MCC and AUC, supported by a relatively high Pearson correlation value 0.928, such trend is dramatically changing if we consider only the experiments with positive MCC and AUC larger than one half. In these cases, Pearson correlation value drops down to 0.803, since the range of possible MCC values for a given AUC value expands almost linearly with increasing AUC, reaching an interval spanning from about 0.2 to 1 for AUC approximating 1. Such data yield that, even for classification tasks whose AUC is very high or almost optimal, the range of possible situations in terms of MCC can be dramatically diverse, and even far from being evaluated as a good model. These cases happen frequently when the dataset is heavily unbalanced, and few samples of the less represented class keep being misclassified: this has an almost negligible effect on the AUC (which results quite high), while is correctly penalized by the MCC, whose value results low.

**Fig. 9:**
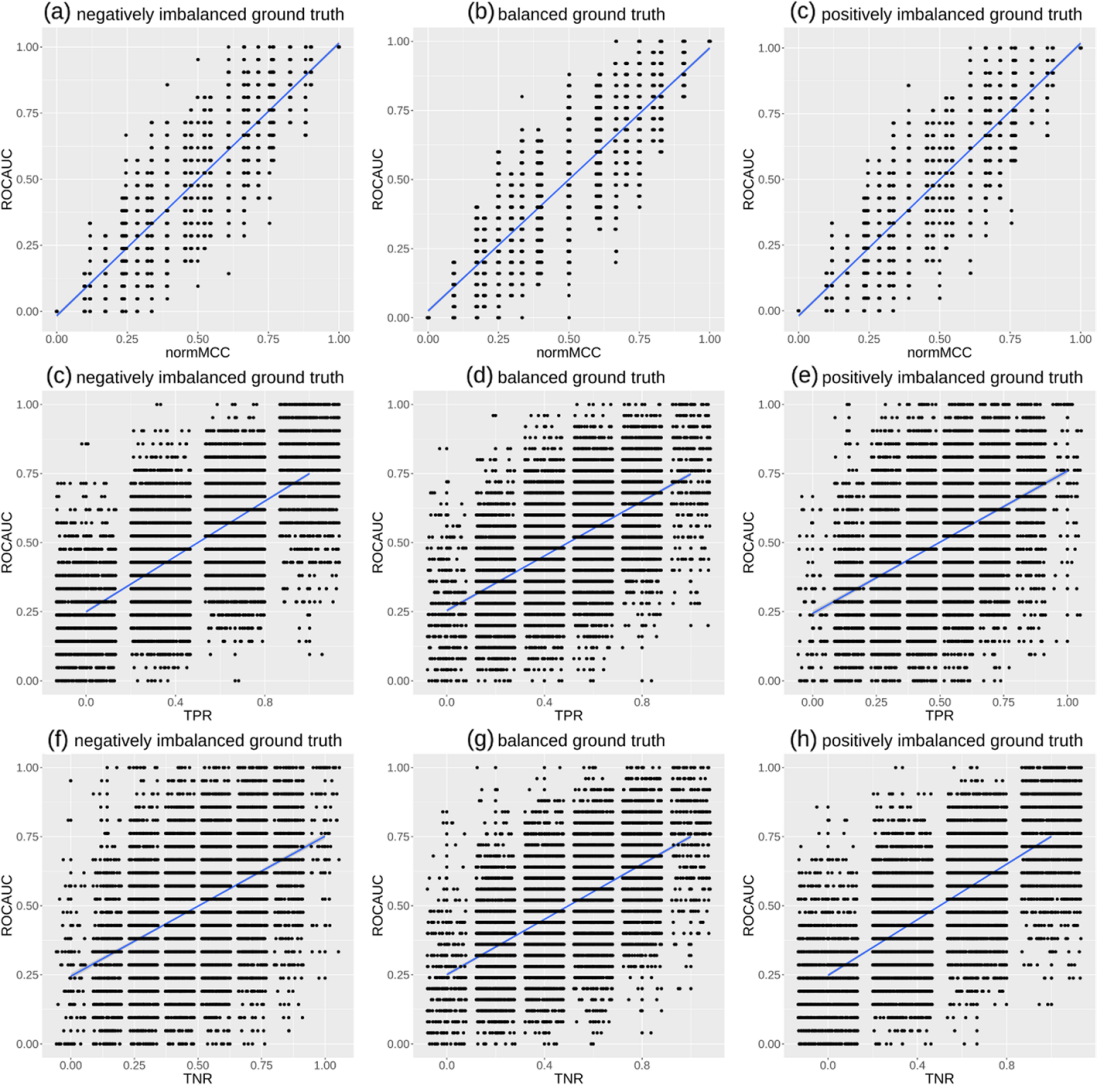
MCC versus ROC AUC plots, TPR versus ROC AUC plots, and TNR versus ROC AUC plots. We developed an R script where we randomly generated a binary ground truth vector of 10 elements, and then we executed a loop where we produced a list of synthesized predictions of real values between 0 and 1, for 10,000 times. For each prediction, we computed the ROC AUC and its corresponding normalized MCC, where $normMCC = (MCC + 1) / 2$, sensitivity, and specificity with cut-off threshold $\tau = 0.5$. Negatively imbalanced ground truth (**a**,**c**,**f**): the ground truth labels are (0, 0, 0, 0, 0, 0, 0, 1, 1, 1), corresponding to 70% negative elements and 30% positive elements. Balanced ground truth (**b**,**d**,**g**): the ground truth labels are (0, 0, 0, 0, 0, 1, 1, 1, 1, 1), corresponding to 50% negative elements and 50% positive elements. Positively imbalanced ground truth (**c**,**e**,**h**): the ground truth labels are (0, 0, 0, 1, 1, 1, 1, 1, 1, 1), corresponding to 30% negative elements and 70% positive elements. In each plot, the ground truth is fixed and never changes, while our script generated 10 random real values in the [0; 1] interval 10,000 times: each time, our script calculates the resulting ROC AUC and normMCC, which corresponds to a single point in the plot. The ground truth values and the predictions are the same of Fig. 10. TPR: true positive rate, sensitivity, recall (Eq. 1). TNR: true negative rate, specificity (Eq. 2). ROC AUC: area under the receiver operating characteristics curve. MCC: Matthews correlation coefficient (Eq. 7). normMCC: normalized MCC (Eq. 8). ROC AUC, normMCC, specificity, and sensitivity range from 0 (minimum and worst value) to 1 (maximum and best value). Blue line: regression line made with smoothed conditional means

**Two-rates plots** In a similar but slightly different approach, we plot hereafter the values generated by ROC AUC and normalized MCC for three ground truths (positively imbalanced, balanced, and negatively imbalanced) of 10 points and 10 thousand different real-valued predictions for the same number of points in Fig. 9.

The three (a, b, c) plots are similar, and show the same trend: ROC AUC and MCC roughly follow the $x = y$ trend, occupying approximately the $x \pm 0.3 = y \pm 0.3$ space. As one can notice, multiple values of ROC AUC correspond to the same Matthews correlation coefficient and vice versa. There are no points near the top left and bottom right corners of the plots, indicating that MCC and ROC are never opposite. However, there are many points for normMCC $\approx$ 0.5 and ROC AUC $\approx$ 0.5, indicating that ROC AUC can have high or low values while MCC indicate a prediction similar to random guessing, and vice versa.

We also generated plots for MCC versus specificity (Fig. 9c, d, e) and for MCC versus specificity (f, g, h). As one can notice, these six plots contain points that occupy almost all the plot space: each ROC AUC point corresponds to almost all the possible values of sensitivity and specificity, with the only relevant exception of the top left and bottom right corners (where ROC AUC $\approx$ 1, TPR $\approx$ 1, and TNR $\approx$ 1).

**Fig. 10:**
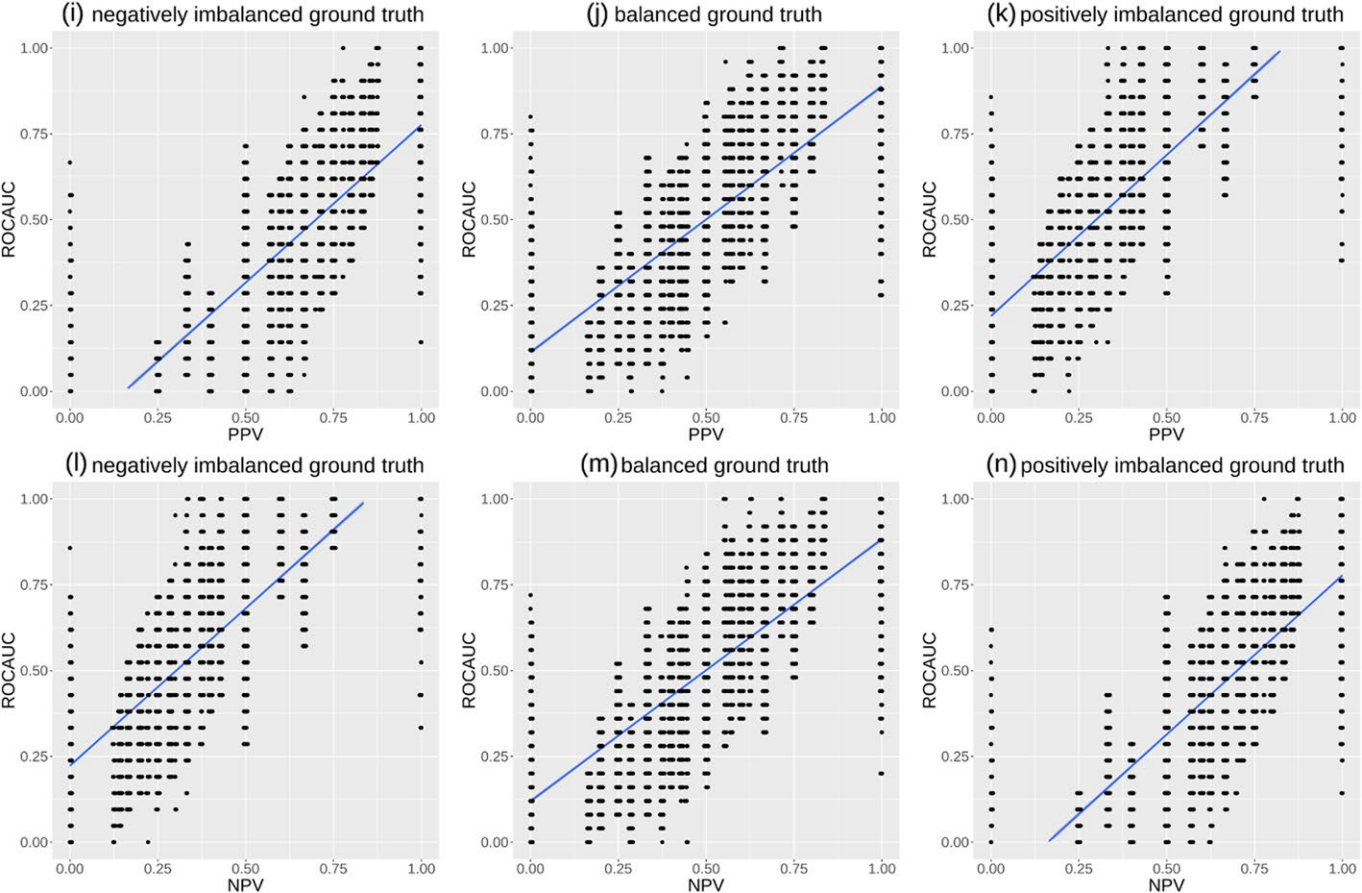
PPV versus ROC AUC plots and NPV versus ROC AUC plots. We developed an R script where we randomly generated a binary ground truth vector of 10 elements, and then we executed a loop where we produced a list of synthesized predictions of real values between 0 and 1, for 10,000 times. For each prediction, we computed the ROC AUC and its corresponding precision (PPV) and negative predictive value (NPV) with cut-off threshold $\tau = 0.5$. Negatively imbalanced ground truth (**i**,**l**): the ground truth labels are (0, 0, 0, 0, 0, 0, 0, 1, 1, 1), corresponding to 70% negative elements and 30% positive elements. Balanced ground truth (**j**,**m**): the ground truth labels are (0, 0, 0, 0, 0, 1, 1, 1, 1, 1), corresponding to 50% negative elements and 50% positive elements. Positively imbalanced ground truth (**k**,**n**): the ground truth labels are (0, 0, 0, 1, 1, 1, 1, 1, 1, 1), corresponding to 30% negative elements and 70% positive elements. In each plot, the ground truth is fixed and never changes, while our script generated 10 random real values in the [0; 1] interval 10,000 times: each time, our script calculates the resulting ROC AUC and normMCC, which corresponds to a single point in the plot. The ground truth values and the predictions are the same of Fig. 9. PPV: precision, positive predictive value (Eq. 3). NPV: negative predictive value (Eq. 4). ROC AUC: area under the receiver operating characteristics curve. ROC AUC, precision, and NPV range from 0 (minimum and worst value) to 1 (maximum and best value). Blue line: regression line made with smoothed conditional means

On the same simulated data, we also produced the ROC AUC versus precision plots and the ROC AUC versus NPV plots (Fig. 10). These plots are similar to the MCC-ROC AUC plots (Fig. 9a, b, c) shown earlier, indicating a common trend between ROC AUC and precision and between ROC AUC and NPV. However, it is clear that here in Fig. 10 the number of points is much less than in the previous cases. Moreover, we can see more points on the ROC AUC axes: precision $=$ 0, NPV $=$ 0, precision $=$ 1, and NPV $=$ 1 correspond to multiple ROC AUCs, including low values and high values. When precision or NPV clearly indicate a bad outcome or a good outcome (values 0 or 1), then ROC AUC can indicate either a poor performance or a good performance. This aspect confirms that ROC AUC is completely uninformative regarding precision and negative predictive value obtained by the classifiers.

## Use cases

To further demonstrate how the MCC is more informative and reliable than the ROC AUC, we list three significant, real use cases of binary classifications obtained through machine learning. We applied Random Forests [58] to three different, independent medical datasets publicly available online:

- UC1: electronic health records of patients with hepatitis C by Tachi et al. [59];
- UC2: electronic health records of patients with chronic kidney disease by Al-Shamsi and coauthors [60];
- UC3: electronic health records of patients with hepatocellular carcinoma by Santos and colleagues [61].

**Fig. 11:**
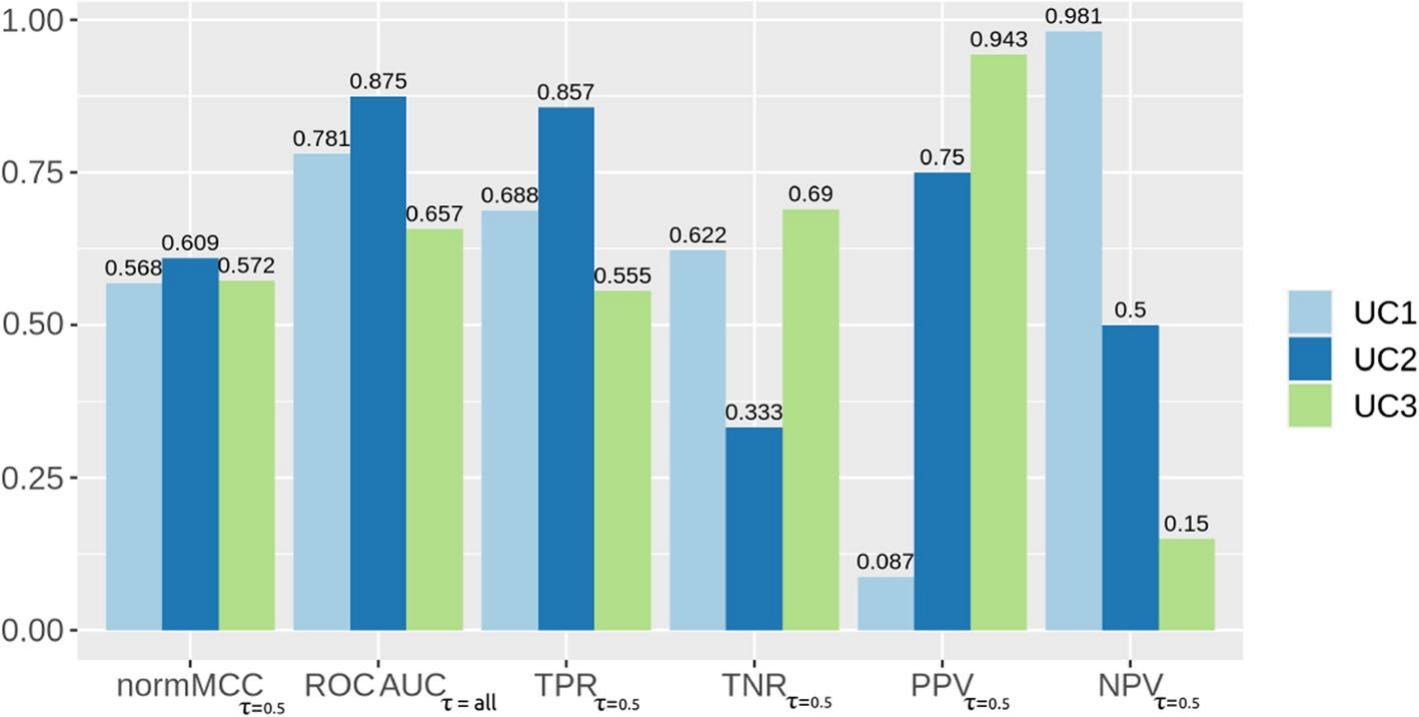
Three use cases including results measured through MCC, ROC AUC, and the four basic rates. Positives: data of survived patients. Negatives: data of deceased patients. MCC: Matthews correlation coefficient. MCC: worst and minimum value $= -1$ and best and maximum value $= +1$. TPR: true positive rate, sensitivity, recall. TNR: true negative rate, specificity. PPV: positive predictive value, precision. NPV: negative predictive value. ROC AUC: area under the receiver operating characteristic curve. The Random Forests classifier generated real predicted values in the [0; 1] interval. For the creation of the ROC curve, we used all the possible $\tau$ cut-off thresholds, as per ROC curve definition. For the creation of the single confusion matrix on which to compute MCC. TPR, TNR, PPV, and NPV, the heuristic traditional $\tau = 0.5$ threshold: predicted values lower than 0.5 were mapped into 0s (negatives), while predicted values greater or equal to 0.5 were mapped into 1s (positives). The resulting positives and negatives were then compared with the ground truth positives and negatives to generate a $\tau = 0.5$ threshold confusion matrix, which we used to calculate the values of MCC. TPR, TNR, PPV, and NPV listed in this table. We report these values in a table format in Table S1. UC1: dataset of electronic health records of patients with hepatitis C by Tachi et al. [59]. UC2: dataset of electronic health records of patients with chronic kidney disease by Al-Shamsi and coauthors [60]. UC3: dataset of electronic health records of patients with hepatocellular carcinoma by Santos and colleagues [61]

We randomly split each dataset into training set (80% patients’ profiles, randomly selected) and test set (20% remaining patients’ profiles), that we used as hold-out validation set [62]. We repeated the execution 100 times and recorded the average value for each final confusion matrix. We reported the results in Fig. 11 and Table S1.

For the UC1 use case, the ROC AUC has a value of almost 0.8, that in the [0; 1] interval means very good classification (Fig. 11 and Table S1). If a researcher decided to only look at this statistic, they would be deceived into thinking that the classifier performed very well. Instead, if we look at the four basic rates, we noticed that the classifier obtained an excellent results for negative predictive value (0.981), sufficient scores for sensitivity and specificity, but an extremely low score for precision (almost zero). The ROC AUC does not reflect the poor performance of the classifier on precision. The MCC, instead, with is low value of +0.135 in the $[-1; +1]$ range, clearly indicates that there is something wrong with this classification. It is clear that the ROC AUC generated an inflated, overoptimistic outcome, while the MCC produced a more reliable result.

In the UC2 use case (Fig. 11 and Table S1), the ROC AUC result being very high: 0.875, almost 0.9, indicating excellent prediction. Again, if a practitioner decided to stop here and not to look at the other rates, they would think their classifier was an excellent one, and that everything went well. The four basic rates, however, tell a different story: while sensitivity and precision are quite high, specificity is quite low and NPV is just sufficient. The ROC AUC fails to communicate the low value of true negative rate. The MCC, instead, with another low value (+0.128), clearly communicates that the classification performance was poor.

In the third use case listed here, UC3 (Fig. 11 and Table S1), we can notice a ROC AUC value of 0.657, indicating a good performance of the classifier. Again, if the researcher stopped here, they would be deceived: the four basic rates tell a different story. A just sufficient sensitivity, a good specificity, an excellent precision, and a very low negative predictive value. The ROC AUC fails to inform us that NPV is almost zero. Again, here the MCC tells us the truth: a low value of +0.144 clearly indicates a poor performance, notifying us that the classifier obtained poor results.

**Recap** In a nutshell, if a study reported the results on these three medical datasets only as area under the receiver operating characteristic curve (ROC AUC $=$ 0.781 for the first use case, ROC AUC $=$ 0.875 for the second use case, and ROC AUC $=$ 0.657 for the third use case, in Table S1), the readership would think that the classifiers performed very well. By looking at the values of sensitivity, specificity, precision, and negative predictive value obtained with the cut-off threshold $\tau = 0.5$, however, one would notice that the classifiers performed poorly on positive predictive value and/or negative predictive value. Conversely, this information is contained in the Matthews correlation coefficient (MCC), whose values correctly inform the readership about the average performance obtained by the classifiers on these datasets.

Warning: even if the MCC is able to correctly advice about the actual poor performance of the classifiers, it does not inform about *why* these performances were poor. To understand why and how the classifiers did not predict efficiently, we recommend binary classification scholars to study the four basic rates  (sensitivity, specificity, precision, and negative predictive value in Table S1).

## Discussion and conclusions

To evaluate binary classifications, it is a common practice to use confusion matrices generated for a particular cut-off threshold. When researchers prefer to consider all the possible thresholds as opposed to picking just one, the rates computed on the confusion matrices can be used as axes for curves, such as the popular and well-known receiver operating characteristic (ROC) curve. A ROC curve has all the possible sensitivity values on the *y* axis and all the possible false positive rate values on the *x* axis; the latter correspond to all the $|1 - specificity|$ scores. A common metric employed in thousands of scientific studies to assess a ROC curve is its area under the curve (AUC), which ranges from 0 meaning completely wrong performance) to 1 meaning perfect performance. The ROC AUC, however, suffers from multiple flaws and pitfalls [33, 34, 37–43] and does not inform us about positive predictive value and negative predictive value. Moreover, as we reported, a high ROC AUC can guarantee only one high value among sensitivity and specificity in the worst case, and high values of both in the best case. Such behavior has its roots in the intrinsic mathematical properties of sensitivity and specificity, the two rates identifying a point in the ROC space. Indeed, not only a point in the ROC space is pointing to multiple given confusion matrices or classes thereof, but to such a given point, the range of MCC values corresponding to the aforementioned ROC point is quite broad, leaving room for a heterogeneous landscape of classifiers, with a quite different set of performances. Consequently, the value of the measure of the area under a ROC curve can represent deeply different situations, which calls into question the ROC AUC reliability as a classifier’s goodness metric. Looking back, it is even surprising that such a faulty metric has been used so frequently in scientific research for so many years, especially for medical decision-making regarding the lives of patients.

Speaking about poor-quality medical research, Douglas G. Altman once wrote: “Once incorrect [medical] procedures become common, it can be hard to stop them from spreading through the medical literature like a genetic mutation” [63, 64]. We believe this to be the case in the usage of ROC curves for binary classification assessment.

In this study, we demonstrated that a more informative and reliable alternative to ROC AUC exists: the Matthews correlation coefficient (MCC). As we explained, a high MCC score always means having high confusion matrix basic rates: sensitivity, specificity, precision, and negative predictive value.

While the MCC has some limitations: it is based on the usage of a heuristic cut-off threshold (usually set at $\tau = 0.5$), and it is undefined in some cases, straightforward mathematical considerations can fill these gaps and allow MCC to be meaningfully defined for all confusion matrices [6]. However, the MCC does not lie: when its value is high, each of the four basic rates of a confusion matrix is high, without exception. This aspect makes the Matthews correlation coefficient superior to the ROC AUC.

As we explained in previous studies [4, 6], the MCC is the most informative and reliable confusion matrix statistic to use if both positive elements and negative elements have the same importance in the scientific study. Only when a researcher wants to give more importance to one group over another, other rates might be more useful. For example, if correctly classifying positive data instances and positive predictions is the main goal of a study, F$_1$ score (Eq. 5) and Fowlkes-Mallows index [65] can be more appropriate rates. In any case, even when one of the two binary categories is more relevant than the other, we recommend to include the MCC among the list of metric employed to assess the results. Moreover, for diagnostics purposes, we suggest to always compute and include not only the MCC, but also the confusion matrix four basic rates (sensitivity, specificity, precision, and negative predictive value): their results can be useful and helpful when a researcher needs to understand *why* their binary classification failed. Broadly speaking, it is always better to employ multiple statistics for results’ evaluation, in any scientific project [66–70]. While the Matthews correlation coefficient can tell *if* the binary classification was unsuccessful, in fact, unfortunately it cannot explain *why*. The four basic rates, on the other hand, can say on which areas of the *predictions versus ground truth* results were problematic.

Even if the four basic rates can be informative, none of them should be used as *the* standard metric to evaluate binary classifications: the MCC should employed for that scope. That is why we here we propose the MCC as the standard rate for binary classification assessments, rather than the ROC AUC.

The ROC curve was invented within the military environment in the 1940s, during the Second World: after more than 80 years of honorable service, we believe its time to retire has come. We have proved that the Matthews correlation coefficient, although less well-known, produces more reliable and more informative results about the correctness or the incorrectness of any binary classification. Therefore we recommend replacing the ROC AUC with the MCC as the standard binary classification metric for any scientific study in any scientific field.TEXCEL

## Supplementary Information

**Additional file 1.**

## Data Availability

Our software code is publicly available under GPL 3.0 license at: https://github.com/davidechicco/MCC_versus_ROC_AUC. The datasets analyzed in the use cases or publically available on FigShare and on the University of California Irvine Machine Learning Repository under the CC BY 4.0 license: • UC1: dataset of electronic health records of patients with hepatitis C by Tachi et al. [59]: https://figshare.com/articles/dataset/_Predictive_Ability_of_Laboratory_Indices_for_Liver_Fibrosis_in_Patients_with_Chronic_Hepatitis_C_after_the_Eradication_of_Hepatitis_C_Virus_/1495100. • UC2: dataset of electronic health records of patients with chronic kidney disease by Al-Shamsi and coauthors [60]: https://figshare.com/articles/dataset/Chronic_kidney_disease_in_patients_at_high_risk_of_cardiovascular_disease_in_the_United_Arab_Emirates_A_population-based_study/6711155?file=12242270. • UC3: dataset of electronic health records of patients with hepatocellular carcinoma by Santos and colleagues [61]: https://archive.ics.uci.edu/ml/datasets/HCC+Survival.

## Abbreviations

AUC: Area under the curve
BM: Bookmaker informedness
CC BY 4.0: Creative Commons Attribution 4.0 International License
CF: Confusion matrix
FN: False negatives
FP: False positives
FPR: False positive rate
MAQC: MicroArray/Sequencing Quality Control
MCC: Matthews correlation coefficient
MK: Markedness
normMCC: Normalized Matthews correlation coefficient
NPV: Negative predictive value
PPV: Positive predictive value, precision
ROC: Receiver operating characteristic
TNR: True negative rate, specificity
TN: True negatives
TPR: True positive rate, sensitivity, recall
TP: True positives
UC: Use case
