# Supplementary material for: The Matthews correlation coefficient (MCC) should replace the ROC AUC as the standard metric for assessing binary classification
Source: BioData Min. 2023 Feb 17;16:4. doi: 10.1186/s13040-023-00322-4 (PMC9938573; doi:10.1186/s13040-023-00322-4)
Supplement: Supplementary file 1 — Additional file 1. [file 13040_2023_322_MOESM1_ESM.pdf]

Supplementary information

**Use case**	**MCC**$_{\tau=0.5}$	**normMCC**$_{\tau=0.5}$	**ROCAUC**$_{\tau=all}$	
UC1	+0.135	0.568	0.781	
UC2	+0.218	0.609	0.875	
UC3	+0.144	0.572	0.657	
range	[−1;+1]	[0; 1]	[0; 1]	
**Use case**	**TPR**$_{\tau=0.5}$	**TNR**$_{\tau=0.5}$	**PPV**$_{\tau=0.5}$	**NPV**$_{\tau=0.5}$
UC1	0.688	0.622	0.087	0.981
UC2	0.857	0.333	0.750	0.500
UC3	0.555	0.690	0.943	0.150
range	[0; 1]	[0; 1]	[0; 1]	[0; 1]

Table S1: ***Three use cases including results measured through MCC, ROCAUC, and the four basic rates***. *Positives: data of survived patients. Negatives: data of deceased patients. MCC: Matthews correlation coefficient. MCC: worst and minimum value* $= -1$ *and best and maximum value* $= +1$. *TPR: true positive rate, sensitivity, recall. TNR: true negative rate, specificity. PPV: positive predictive value, precision. NPV: negative predictive value. ROCAUC: area under the receiver operating characteristic curve. The Random Forests classifier generated real predicted values in the* $[0; 1]$ *interval. For the creation of the ROC curve, we used all the possible* τ *cut-off thresholds, as per ROC curve definition. For the creation of the single confusion matrix on which to compute MCC. TPR, TNR, PPV, and NPV, the heuristic traditional* $\tau = 0.5$ *threshold: predicted values lower than 0.5 were mapped into 0s(negatives), while predicted values greater or equal to 0.5 were mapped into 1s(positives). The resulting positives and negatives were then compared with the ground truth positives and negatives to generate a* $\tau = 0.5$ *threshold confusion matrix, which we used to calculate the values of MCC. TPR, TNR, PPV, and NPV listed in this table. We report these values as barcharts in Figure 11. UC1: dataset of electronic health records of patients with hepatitis C by Tachietal. [59]. UC2: dataset of electronic health records of patients with chronic kidney disease by Al-Shamsiandcoauthors [60]. UC3: dataset of electronic health records of patients with hepatocellular carcinoma by Santosandcolleagues [61].*
